# Associations between commute mode use and self-rated health and work ability among Finnish public sector employees

**DOI:** 10.1177/14034948231159212

**Published:** 2023-03-20

**Authors:** Essi Kalliolahti, Ville Aalto, Paula Salo, Timo Lanki, Jenni Ervasti, Tuula Oksanen

**Affiliations:** 1 University of Eastern Finland, Institute of Public Health and Clinical Nutrition, Kuopio, Finland; 2 Finnish Institute of Occupational Health, Helsinki, Finland; 3 University of Turku, Department of Psychology and Speech-Language Pathology, Turku, Finland; 4 Finnish Institute for Health and Welfare (THL), Environmental Health, Kuopio, Finland; 5 University of Eastern Finland, Department of Environmental and Biological Sciences, Kuopio, Finland

**Keywords:** Active commuting, commute mode, self-rated health, work ability, occupational health

## Abstract

**Aim:**

To determine the extent to which level of active commute mode use is associated with self-rated health and work ability.

**Methods:**

The data were sourced from the Finnish Public Sector Study survey in 2020 (*n* = 38,223). The associations between active commuting – assessed with the frequency of using active commute modes – and self-rated health and work ability were examined with negative binomial regression analyses. Passive commuting and low-to-moderate levels of active commuting were compared with active commuting, and the models were adjusted for sociodemographic factors, working time mode, and lifestyle risk factors. We also assessed separate associations between walking and cycling as a mode of commuting by additionally considering the commuting distance and the outcomes.

**Results:**

After adjustment, when using active commuters as a reference, passive commuters had a 1.23-fold (95% confidence intervals (CI) 1.19 to 1.29) risk of suboptimal self-rated health and a 1.18-fold (95% CI 1.13 to 1.22) risk of suboptimal work ability. More frequent and/or longer distance by foot and especially by bicycle, was positively associated with health and work ability. Never commuting by bicycle was associated with a 1.65-fold (95% CI 1.55 to 1.74) risk of suboptimal health and a 1.27-fold (95% CI 1.21 to 1.34) risk of suboptimal work ability when using high-dose bicycle commuting as a reference.

**Conclusions:**

**Passive commuting was associated with suboptimal self-rated health and suboptimal work ability. Our results suggest that using active commute modes, particularly cycling, may be beneficial for employee health and work ability.**

## Introduction

Regular physical activity maintains and promotes physical and mental health. For adults aged 18 to 64, the World Health Organization recommends moderate-intensity physical activity for at least 150 min per week, but worldwide almost one in four adults does not meet these recommendations [1]. In Finland, the corresponding proportion of physically inactive adults is around 50% [2]. Active commuting to and from work, such as walking or cycling, may be a feasible way of increasing the levels of weekly physical activity and help people to meet the global recommendations.

Previous studies suggest that active commuting reduces the risk of several non-communicable diseases and conditions such as cardiovascular diseases [3-6], cancer [3,6], diabetes [4,7], obesity and overweight [8,9], and all-cause mortality [3-6]. A recent meta-analysis of prospective cohort studies found that walking and cycling for the commute was associated with an 8% lower risk of cardiovascular disease incidence (i.e. coronary heart disease, stroke and heart failure), a 9% lower risk of all-cause mortality, and a 30% lower risk of diabetes [4]. All active travel modes, including mixed modes such as a combination of public transport and walking or cycling, have been associated with health benefits, yet the associations vary depending on the mode [3,4,6,10].

While studies on the health benefits of active commuting exist, studies on the associations between commute mode use and employee self-rated health and especially work ability are limited. Previous studies have found associations with active travel in general and better self-rated health [8,10], and with active commuting in particular and higher levels of subjective physical health and wellbeing [11,12]. Additionally, previous literature suggests that leisure-time physical activity is associated with better work ability [13-16] and that active commuting contributes to total physical activity rather than being a substitute for other leisure-time physical activity [17,18]. Supported by the literature, we hypothesised that a higher level of active commuting is associated with both optimal self-rated health and work ability.

The main aim of this study was to examine whether the frequency of commuting by active commute modes was associated with self-rated health and work ability among public sector employees in Finland. We aimed to provide a more realistic description of active commuting behaviour than in most previous studies by considering that people may use multiple active commute modes with varying frequencies on a regular basis, which all contributes to weekly physical activity.

## Methods

### Study design and participants

This cross-sectional questionnaire study was nested within the ongoing prospective Finnish Public Sector (FPS) Study [19]. Study participants were FPS employees working in the service of the Southern Finland cities of Helsinki, Espoo, Vantaa and Turku in 2020. The questionnaire included questions on commuting behaviour and was sent to 58,971 public sector employees and 42,574 responded (response rate 72%). The sample represented about 10% of the entire public sector personnel in Finland in 2020. Of these, 318 did not report any information on commuting behaviour, self-rated health and/or work ability, and thus they were excluded. Of the remaining 42,256 participants, an additional 4033 were excluded because they had missing data for any one or more of the potential confounders: sex, age, occupational status, marital status, working time mode, smoking status, and alcohol use. The final analytic sample (*n* = 38,223) consisted of participants with information on all relevant variables.

The average age of the participants was 46 years, and the majority (78%) of them were women. The most common occupations were lecturers/subject teachers (*n* = 4425) followed by health and social care workers, namely practical/assistant nurses (*n* = 3812), registered nurses/public health nurses (*n* = 2989) and social counsellors/youth workers (*n* = 2962).

### Active commuting

We asked participants to evaluate how often they use each commute mode separately for ‘summer and winter conditions’. Commute modes were (1) walking, (2) cycling, (3) public transport with 1000 m or more of walking or cycling, (4) public transport with less than 1000 m of walking or cycling, and (5) private car use, either as a driver or a passenger. Of these categories, 1–3 were considered as active commute modes and 4 and 5 as passive commute modes. Response categories were 1 = daily or almost daily; 2 = a few times a week; 3 = once a week; 4 = less than once a week; 5 = never. If the participant responded to at least one commute mode use during each season but not all, missing values were imputed with ‘never’.

We created a scoring method for assessing the level of active commuting of the participants: daily or almost daily = 5 points; a few times a week = 3 points; once a week = 1 point; less than once a week = 0.5 points; and never = 0 points. The scoring was based on the frequency (days per week) a participant was approximately expected to use the mode. Passive commute modes were not scored any points. Points gained from active commuting during summer and winter were added up and 10 points was set as the maximum score, although our scoring method allowed the accumulation of more points than this. For statistical analyses, level of active commuting was further categorised into passive (= 0 points), low to moderate (= 0.5–9.5 points), and active (= 10 points). The unequal distribution of the continuous variable determined, in part, the selected threshold values for the categories.

We also separately assessed the ‘dose’ of walking and cycling to work by additionally considering the commuting distance. Participants were scored according to their use of the specific mode and the scores from summer and winter were added up as in our main model. Next, the scores were multiplied by their reported one-way commuting distance (km). Further, participants were categorised into three groups based on the multiplication: never (= 0), low to moderate (= median or less), and high-dose (= higher than median) commuting by the mode. The median value for walking was 9.1 (mean distance = 3.7 km; standard deviation (SD) = 6.2), and for cycling it was 16.5 (mean distance = 7.1 km; SD = 7.5).

### Self-rated health

Self-rated health was measured with a single item question ‘How do you rate your health?’ [20]. Despite the subjective nature of self-rated health, it is, in fact, a strong predictor of morbidity [21] and mortality [21,22]. Response options were 1 = good; 2 = fairly good; 3 = average; 4 = fairly poor; and 5 = poor. For statistical analyses, self-rated health was dichotomised into optimal (= 0) and suboptimal (= 1) in a way that optimal was comprised of the response options ‘good’ and ‘fairly good’ and suboptimal of ‘average’, ‘fairly poor’ and ‘poor’.

### Self-rated work ability

Self-rated work ability was measured using a single item from the Work Ability Index (WAI) [23]: ‘Let’s assume that your work ability at its all-time best would be given 10 points, and 0 points would indicate that you are completely unable to work. How would you score your current work ability?’. This single question measure is based on the use of the first item of the WAI’s seven-item questionnaire, which is also referred to as the Work Ability Score (WAS). WAS has been shown to be a similarly valid but simpler alternative to WAI [24], which is a subjective estimation of an employee’s work ability in relation to the health status and resources of an employee and the work demands [23]. For statistical analyses, self-rated work ability was dichotomised into optimal (= 0) and suboptimal (= 1) in a way that response options of 8 and above were considered to be optimal and of 7 or less were considered suboptimal [24].

### Covariates

We controlled for sex, age (continuous), occupational status, marital status, working time mode, smoking status, and alcohol use. Participants were categorised into three socioeconomic status levels according to the 2001 International Standard Classification of Occupations codes: high (managers and senior specialists such as physicians and teachers), intermediate (specialists, office workers and customer service and health and social care workers) and low (manual workers including construction and cleaning services workers and for example, practical nurses. Working time mode was dichotomised into regular daytime work and other work (shift, non-regular daytime, and regular night work). Smoking was dichotomised into non-smokers (never and former smokers) and current smokers and alcohol use into no use or moderate use and at-risk use. At-risk alcohol use for women was more than 11 units or 140 g and for men more than 23 units or 280 g of alcohol per week. One unit was approximately equivalent to one drink or one glass of alcoholic drink or 12 g of alcohol [25].

## Statistical analyses

The descriptive statistics of our study population are presented as frequencies and percentages for categorical variables and means and SDs for age as the only continuous variable. To compare differences in the distribution of characteristics, the chi-square test was used for categorical variables and a one-way analysis of variance for age. We used log-binomial regression to study the associations between the level of active commuting and the outcomes. Active commuters were used as a reference. A log-binomial model is a generalised linear model with a logarithmic link function and a binomial distribution function producing relative risk estimates (RR) with their 95% CI. In Model 1, we tested the bivariate association between active commuting and the outcome. Model 2 included Model 1 and the following sociodemographic factors: sex, age, occupational status and marital status. In Model 3, we further added working time mode and lifestyle factors (smoking status and alcohol use). We also assessed separate associations between walking and cycling as a mode of commuting by additionally considering the commuting distance and the outcomes. Adjustments were made as in our main models.

The Statistical Package for the Social Sciences (SPSS) version 27 (IBM SPSS Statistics for Windows, IBM Corp., New York, NY, USA) was used for statistical analyses. The SAS software package (V.9.4; SAS Institute) was used for data management.

## Results

The distribution of outcome variables and other key variables by the level of active commuting are presented in Table I. A correlation matrix (Spearman’s rank correlation coefficients) for all variables before dichotomisation of the outcome variables is presented in Supplemental Table S1. Commute mode shares for summer and winter separately by the level of mode use (frequencies and percentages) are presented in Supplemental Table S2.

**Table I.** The characteristics of the study population by level of active commuting. Values are counts and percentages unless otherwise stated.

Variables	Total (*n*= 38,223)	Passive (*n* = 12,707)	Low to moderate (*n* = 11,445)	Active (*n* = 14,071)	*P* for difference
Sex					
Women	29,948 (78%)	9779 (77%)	8587 (75%)	11,582 (82%)	
Men	8275 (22%)	2928 (23%)	2858 (25%)	2498 (18%)	<0.001
Age, mean (SD)	46.0 (11.0)	46.8 (10.8)	45.5 (10.7)	45.6 (11.3)	<0.001
Socioeconomic status					
High	19,536 (51%)	6142 (48%)	6167 (54%)	7227 (52%)	
Intermediate	10,597 (28%)	3706 (29%)	3046 (27%)	3845 (27%)	
Low	8090 (21%)	2859 (23%)	2232 (20%)	2999 (21%)	<0.001
Marital status					
Unmarried	11,545 (30%)	3535 (28%)	3050 (27%)	4960 (35%)	
Married	18,898 (49%)	6652 (52%)	6058 (53%)	6188 (44%)	
Cohabitation (yes)	7780 (20%)	2520 (20%)	2337 (20%)	2923 (21%)	<0.001
Working time mode					
Regular daytime	30,181 (79%)	10,002 (79%)	8949 (78%)	11,230 (80%)	
Other	8042 (21%)	2705 (21%)	2496 (22%)	2841 (20%)	0.005
Smoking					
No	33,879 (89%)	10,884 (86%)	10,418 (91%)	12,577 (89%)	
Yes	4344 (11%)	1823 (14%)	1027 (9%)	1494 (11%)	<0.001
Alcohol use					
No use/ moderate use	36,599 (96%)	12,124 (95%)	10,992(96%)	13,483 (96%)	
At-risk use	1624 (4%)	583 (5%)	453 (4%)	588 (4%)	0.046
Self-rated health					
Suboptimal	8997 (24%)	3509 (28%)	2405 (21%)	3083 (22%)	
Optimal	29,226 (77%)	9198 (73%)	9040 (79%)	10,988 (78%)	<0.001
Work ability					
Suboptimal	10,542 (28%)	3910 (31%)	2991 (26%)	3641 (26%)	
Optimal	27,681 (72%)	8797 (69%)	9454 (74%)	10,430 (74%)	<0.001

Active commuting was associated with self-rated health and work ability. In models adjusted for sociodemographic and lifestyle risk factors, passive commuters had a 1.23-fold (95% CI 1.19 to 1.29) risk of suboptimal self-rated health and a 1.18-fold (95% CI 1.13 to 1.22) risk of suboptimal work ability compared with active commuters (Table II).

**Table II.** Associations between active commuting and suboptimal self-rated health and work ability.

	Suboptimal self-rated health			Suboptimal work ability		
	Model 1*	Model 2**	Model 3***	Model 1*	Model 2**	Model 3***
Level of active commuting	RR (95% CI)	RR (95% CI)	RR (95% CI)	RR (95% CI)	RR (95% CI)	RR (95% CI)
Active (reference)	1	1	1	1	1	1
Low to moderate	0.96 (0.92 to 1.01)	1.01 (0.97 to 1.06)	1.01 (0.97 to 1.06)	1.01 (0.97 to 1.05)	1.04 (1.00 to 1.08)	1.04 (1.00 to 1.08)
Passive	1.26 (1.21 to 1.31)	1.25 (1.20 to 1.30)	1.23 (1.19 to 1.29)	1.19 (1.15 to 1.24)	1.19 (1.14 to 1.23)	1.18 (1.13 to 1.22)

* Model 1 is unadjusted.

** Model 2 is adjusted for sex, age, occupational status, and marital status.

*** Model 3 is adjusted as Model 2 and additionally for working time mode, smoking, and at-risk alcohol use.

In the analyses additionally considering commuting distance (i.e. ‘dose’ of commuting by foot or bicycle), never commuting by foot was associated with a 1.11-fold (95% CI 1.05 to 1.18) risk of suboptimal self-rated health and a 1.08-fold (95% CI 1.03 to 1.14) risk of suboptimal work ability compared with high-dose walk commuting after adjustments for sociodemographic and lifestyle risk factors (Table III). After adjustment, never commuting by bicycle was associated with a 1.65-fold (95% CI 1.55 to 1.74) risk of suboptimal self-rated health and a 1.27-fold (95% CI 1.21 to 1.34) risk of suboptimal work ability compared with high-dose bicycle commuting. For bicycle users, the associations seemed to follow a dose–response pattern. (Table IV).

**Table III.** Associations between walking as a mode of commuting and suboptimal self-rated health and work ability.

	Suboptimal self-rated health			Suboptimal work ability		
	Model 1*	Model 2**	Model 3***	Model 1*	Model 2**	Model 3***
‘Dose’ of walking	RR (95% CI)	RR (95% CI)	RR (95% CI)	RR (95% CI)	RR (95% CI)	RR (95% CI)
High (reference) (*n* = 4587)	1	1	1	1	1	1
Low to moderate (*n* = 4826)	1.03 (0.95 to 1.11)	1.06 (0.99 to 1.14)	1.05 (0.98 to 1.13)	1.00 (0.93 to 1.07)	1.02 (0.95 to 1.09)	1.01 (0.95 to 1.08)
Never (*n* = 28,357)	1.07 (1.01 to 1.13)	1.12 (1.06 to 1.19)	1.11 (1.05 to 1.18)	1.05 (1.00 to 1.11)	1.08 (1.03 to 1.14)	1.08 (1.03 to 1.14)

* Model 1 is unadjusted.

** Model 2 is adjusted for sex, age, occupational status, and marital status.

*** Model 3 is adjusted as Model 2 and additionally for working time mode, smoking, and at-risk alcohol use.

**Table IV.** Associations between bicycle commuting and suboptimal self-rated health and work ability.

	Suboptimal self-rated health			Suboptimal work ability		
	Model 1*	Model 2**	Model 3***	Model 1*	Model 2**	Model 3***
‘Dose’ of cycling	RR (95% CI)	RR (95% CI)	RR (95% CI)	RR (95% CI)	RR (95% CI)	RR (95% CI)
High (reference) (*n* = 7280)	1	1	1	1	1	1
Low to moderate (*n* = 7818)	1.40 (1.31 to 1.50)	1.35 (1.26 to 1.45)	1.34 (1.25 to 1.44)	1.15 (1.08 to 1.22)	1.13 (1.06 to 1.19)	1.12 (1.06 to 1.18)
Never (*n* = 22,710)	1.87 (1.77 to 1.99)	1.67 (1.58 to 1.78)	1.65 (1.55 to 1.74)	1.37 (1.31 to 1.44)	1.29 (1.23 to 1.35)	1.27 (1.21 to 1.34)

* Model 1 is unadjusted.

** Model 2 is adjusted for sex, age, occupational status, and marital status.

*** Model 3 is adjusted as Model 2 and additionally for working time mode, smoking, and at-risk alcohol use.

## Discussion

In this study, we examined whether the level of active commuting, assessed with a composite score on active commute mode use, was associated with self-rated health and work ability among FPS employees. We found that after adjusting for sociodemographic factors, working time mode, and lifestyle factors, the RR of suboptimal self-rated health was higher among those employees who never commuted by any of the active commute modes compared with active commuters.

Our findings are in line with some previous studies [8,10-12]. A cross-sectional study in an adult Swedish population [8] found that passive travel was associated with a greater risk of poor self-rated health compared with active travel, and evidence from seven European cities [10] showed that more frequent walking and cycling for transportation was associated with better self-rated health. Furthermore, longitudinal evidence from the UK suggests that switching from car commuting to active commute mode use has a positive effect on perceived physical health among women, and that switching from active commuting to car use has a negative effect on perceived physical health and health satisfaction among both sexes [12]. In general, it may be that the health benefits of active commuting stem not only from the commensurate increase in total physical activity [17,18], but also from the substitution of passive commute modes. We did not, however, find a dose–response relationship between the level of active commuting and self-rated health. This may be due to our three-category scoring method in which the group of low- to moderate-activity-level commuters also included participants with such low-frequency active commuting that it did not differ much from being a passive commuter.

We are not aware of previous studies on the associations between active commuting in particular and work ability. We found that employees only using passive commute modes had a higher RR of suboptimal work ability compared with more active commuters, which suggests that active commute mode use is associated with better work ability. This is supported by the strong scientific evidence on the positive associations between leisure-time physical activity in general and perceived work ability [13-16]. Among the working population, leisure-time physical activity may reduce musculoskeletal symptoms [15,26], especially low back pain among sedentary workers [26]. Overall, there is little doubt that people with sedentary jobs benefit from engaging in more leisure-time physical activity such as active commuting, but a higher level of active commuting may also help employees to achieve a balance between the resources and physical work demands in occupations with high occupational physical activity. In a previous cross-sectional study among Finnish men, the positive relationship between recreational physical activity and work ability was, in fact, stronger in more physically demanding jobs [16].

Based on our additional analyses, more frequent and/or longer distance (i.e. higher ‘dose’) bicycle commuting appeared to be more strongly associated with optimal self-rated health and work ability than walking. Our findings are in line with previous studies in which the associations between cycling and physical health outcomes provided the most robust results [3,4,6,10]. Yet, our results suggest that also commuting regularly by foot may have the potential to positively affect employee health and work ability. This finding is of high relevance because for many, walking is likely to be more accessible than cycling and it does not require equipment. Regular bicycle commuting (especially long-distance) may also require at least some commitment, which may result from a higher commitment to an active and healthier lifestyle in general and thus, there is a more reasonable possibility of reverse causation.

The greatest strengths of our study lie in the substantial number of participants and the representativeness of the FPS workforce. The FPS study, within which this study was nested, is the most comprehensive and long-running study on public sector employees’ health and wellbeing in Finland. Due to the survey questionnaire design and scoring the level of each active commute mode use, we were also able to capture a diverse range of active commuting behaviour combinations. In our separate analyses on walking and cycling, we were able to consider the frequency of commuting in relation to commuting distance and to increase the robustness of our main findings. Moreover, to our knowledge, the associations between commute mode use and work ability have not been previously studied.

Our study has some limitations to be noted. First, the cross-sectional design does not allow us to draw any firm conclusions on the causal relationship between active commuting and the outcomes nor to determine the direction of causation. It is possible that people who perceive their health and work ability to be optimal are more inclined to use active commute modes than those with suboptimal self-rated health and work ability. Second, we lacked information on commuting time, and we were not able to adjust our models for leisure-time- and work-related physical activities. The dichotomisation of outcome variables may also involve some disadvantages [27]. However, it is often useful to create a cut point for simpler interpretation, and to stratify population according to risk when planning interventions.

When constructing our composite score on active commute mode use, we did not distinguish between walking and cycling although walking is, usually, less energy-intensive per unit of time than cycling [28]. To gain health benefits from commuting by foot may require more time than people are able or willing to spend on travelling to and from work. However, in our additional analyses on the ‘dose’ of active commuting, we did separate walking and cycling, and our results corroborated those of earlier studies. Further, we included commuting by public transport with 1000 m or more walking or cycling in active commute modes, but we lacked information on whether this was done by walking or cycling and how much of the commute was undertaken actively. Because of this, we were not able to examine active public transport in relation to commuting distance. A prospective cohort study from the UK [3] found that 90% of solely cycling- and 80% of mixed mode cycling commuters met the current physical activity guidelines. Instead, among solely walking- and mixed-mode walking commuters the corresponding proportions were 54% and 50%, respectively, which did not differ much from the proportions among passive commuters [3]. However, a significant amount of physical activity can be achieved through commuting by public transport with walking, especially among rail commuters who often walk longer distances than bus commuters [29], but we also lacked information on the type of public transport. Public transport itself may also have opposite health effects compared with walking and cycling, and not only because it is a passive commute mode. In a Swedish study, long commute time when using public transport (or private car) was found to be associated with poor self-rated health and adverse health effects such as sleep disorders, everyday stress and low vitality [30]. Because the health effects of active commuting are likely to vary depending not only on the mode, frequency and distance, but also on the duration, intensity and possible other factors, more detailed research is needed. Also, use of more marginal commute modes such as motorbikes, electric scooters, and e-bikes was not included in this study. Finally, the data were self-reported and, as a result, information bias may exist.

## Conclusions

Active commute mode use, particularly cycling, is associated with better self-rated health and work ability among the public sector workforce. Longitudinal and intervention studies are needed to demonstrate whether the promotion of active commuting could improve employee health and work ability.

## Supplemental Material

sj-docx-1-sjp-10.1177_14034948231159212 – Supplemental material for Associations between commute mode use and self-rated health and work ability among Finnish public sector employees Supplemental material, sj-docx-1-sjp-10.1177_14034948231159212 for Associations between commute mode use and self-rated health and work ability among Finnish public sector employees by Essi Kalliolahti, Ville Aalto, Paula Salo, Timo Lanki, Jenni Ervasti and Tuula Oksanen in Scandinavian Journal of Public Health
