# Supplementary material for: Associations between commute mode use and self-rated health and work ability among Finnish public sector employees
Source: Scand J Public Health. 2023 Mar 20;52(4):468–75. doi: 10.1177/14034948231159212 (PMC11179311; doi:10.1177/14034948231159212)
Supplement: sj-docx-1-sjp-10.1177_14034948231159212 – Supplemental material for Associations between commute mode use and self-rated health and work ability among Finnish public sector employees [file sj-docx-1-sjp-10.1177_14034948231159212.docx]

**Table S1. Spearman´s rank correlation coefficients for all covariate and outcome variables.**

|  | 1 | 2 | 3 | 4 | 5 | 6 | 7 | 8 | 9 |
| --- | --- | --- | --- | --- | --- | --- | --- | --- | --- |
| 1 Sex (1=women; 2=men) | 1 |  |  |  |  |  |  |  |  |
| 2 Age (years) | -0.004 | 1 |  |  |  |  |  |  |  |
| 3 SES (1=high; 2=intermediate; 3=low) | 0.20** | -0.012* | 1 |  |  |  |  |  |  |
| 4 Marital status (1=unmarried; 2=married; 3=cohabitant) | 0.048** | -0.109** | -0.055** | 1 |  |  |  |  |  |
| 5 Working time mode (1=regular daytime; 2=other) | 0.084** | -0.078** | 0.496** | -0.040** | 1 |  |  |  |  |
| 6 Smoking (1=non-smoker; 2=smoker) | 0.012* | -0.007 | 0.182** | -0.059** | 0.141** | 1 |  |  |  |
| 7 Alcohol use (1=no/moderate use; 2=at-risk use) | -0.001 | 0.073** | -0.023** | -0.011* | -0.017** | 0.082** | 1 |  |  |
| 8 Self-rated health (1–5; 1=good; 5=poor) | 0.020** | -0.197** | -0.119** | -0.063** | -0.059** | -0.093** | 0.054** | 1 |  |
| 9 Work ability (0–10; 0=completely unable to work;10=all-time best) | 0.016** | -0.140** | -0.089** | 0.046** | -0.064** | -0.062** | -0.042** | -0.705** | 1 |

**Statistically significant at the 0.001 level

*Statistically significant at the 0.05 level

**Supplemental Table S2. Commute mode shares for summer and winter by the frequency of using the mode.** Values are counts and percentages.

|  | **Daily or almost daily** | **A few times a week** | **Once a week** | **Less than once a week** | **Never** |
| --- | --- | --- | --- | --- | --- |
| **Walking**  Summer/  Winter | 3363 (9.8%)/  4153 (10.9%) | 1336 (3.5%)/  1533 (4.0%) | 766 (2.0%)/  769 (2.0%) | 3122 (8.2%)/  2304 (6.0%) | 29615 (77.5%)/  29414 (77%) |
| **Cycling**  Summer/  Winter | 7240 (18.9%)/  2317 (6.1%) | 3181 (8.3%)/  1541 (4.0%) | 1266 (3.3%)/  626 (1.6%) | 3752 (9.8%)/  1694 (4.4%) | 22763 (59.6%)/  31995 (83.7%) |
| **Active public transport** Summer/  Winter | 5675 (14.8%)/  7375 (19.3%) | 2128 (5.6%)/  2458 (6.4%) | 1019 (2.7%)/  919 (2.4%) | 3774 (9.9%)/  2834 (7.4%) | 25606 (67%)/  24587 (64.3%) |
| **Passive public transport** Summer/  Winter | 4311 (11.3%)/  5417 (14.2%) | 1576 (4.1%)/  1811 (4.7%) | 755 (2.0%)/  656 (1.7%) | 2855 (7.5%)/  2223 (5.8%) | 28705 (75.1%)/  28066 (73.4%) |
| **Driving**  Summer/  Winter | 14366 (37.6%)/  15428 (40.4%) | 3359 (8.8%)/  3332 (8.7%) | 1425 (3.7%)/  1209 (3.2%) | 3334 (8.7%)/  2889 (7.6%) | 15718 (41.1%)/  15315 (40.1%) |
